# Znf179 E3 ligase-mediated TDP-43 polyubiquitination is involved in TDP-43- ubiquitinated inclusions (UBI) (+)-related neurodegenerative pathology

**DOI:** 10.1186/s12929-018-0479-4

**Published:** 2018-11-08

**Authors:** Yi-Chao Lee, Wan-Chen Huang, Jiann-Her Lin, Tzu-Jen Kao, Hui-Ching Lin, Kuen-Haur Lee, Hsin-Chuan Lin, Che-Kun James Shen, Wen-Chang Chang, Chi-Chen Huang

**Affiliations:** 1 Graduate Institute of Neural Regenerative Medicine, College of Medical Science and Technology/Center for Neurotrauma and Neuroregeneration, Taipei Medical University, Taipei, 115 Taiwan; 2 Institute of Cellular and Organismic Biology, Academia Sinica, Taipei, 115 Taiwan; 3 Department of Neurosurgery, Taipei Medical University, Taipei, Taiwan; 4 Division of Neurosurgery, Department of Surgery, School of Medicine, College of Medicine, Taipei Medical University, Taipei, Taiwan; 5 Institute and Department of Physiology, School of Medicine, National Yang-Ming University, Taipei, 112 Taiwan; 6 Graduate Institute of Cancer Biology and Drug Discovery, College of Medical Science and Technology, Taipei Medical University, Taipei, 115 Taiwan; 7 Graduate Institute of Medical Sciences, College of Medicine, Taipei Medical University, Taipei, 110 Taiwan; 8 Institute of Molecular Biology, Academia Sinica, Taipei, 115 Taiwan

**Keywords:** Znf179, TDP-43, Polyubiquitination, E3 ligase, ALS, FTLD-U

## Abstract

**Background:**

The brain predominantly expressed RING finger protein, Znf179, is known to be important for embryonic neuronal differentiation during brain development. Downregulation of Znf179 has been observed in motor neurons of adult mouse models for amyotrophic lateral sclerosis (ALS), yet the molecular function of Znf179 in neurodegeneration has never been previously described. Znf179 contains the classical C3HC4 RING finger domain, and numerous proteins containing C3HC4 RING finger domain act as E3 ubiquitin ligases. Hence, we are interested to identify whether Znf179 possesses E3 ligase activity and its role in ALS neuropathy.

**Methods:**

We used in vivo and in vitro ubiquitination assay to examine the E3 ligase autoubiquitination activity of Znf179 and its effect on 26S proteasome activity. To search for the candidate substrates of Znf179, we immunoprecipitated Znf179 and subjected to mass spectrometry (MS) analysis to identify its interacting proteins. We found that ALS/ FTLD-U (frontotemporal lobar degeneration (FTLD) with ubiquitin inclusions)-related neurodegenerative TDP-43 protein is the E3 ligase substrate of Znf179. To further clarify the role of E3 ubiquitin ligase Znf179 in neurodegenerative TDP-43-UBI (ubiquitinated inclusions) (+) proteinopathy, the effect of Znf179-mediated TDP-43 polyubiquitination on TDP-43 protein stability, aggregate formation and nucleus/cytoplasm mislocalization were evaluated in vitro cell culture system and in vivo animal model.

**Results:**

Here we report that Znf179 is a RING E3 ubiquitin ligase which possesses autoubiquitination feature and regulates 26S proteasome activity through modulating the protein expression levels of 19S/20S proteasome subunits. Our immunoprecipitation assay and MS analysis results revealed that the neuropathological TDP-43 protein is one of its E3 ligase substrate. Znf179 interactes with TDP-43 protein and mediates polyubiquitination of TDP-43 in vitro and in vivo*.* In neurodegenerative TDP-43 proteinopathy, we found that Znf179-mediated polyubiquitination of TDP-43 accelerates its protein turnover rate and attenuates insoluble pathologic TDP-43 aggregates, while knockout of Znf179 in mouse brain results in accumulation of insoluble TDP-43 and cytosolic TDP-43 inclusions in cortex, hippocampus and midbrain regions.

**Conclusions:**

Here we unveil the important role for the novel E3 ligase Znf179 in TDP-43-mediated neuropathy, and provide a potential therapeutic strategy for combating ALS/ FTLD-U neurodegenerative pathologies.

**Electronic supplementary material:**

The online version of this article (10.1186/s12929-018-0479-4) contains supplementary material, which is available to authorized users.

## Background

The non-amyloidogenic ubiquitinated inclusions (UBI) are found to be the common neuropathology in both FTLD-U (frontotemporal lobar degeneration (FTLD) and ALS (amyotrophic lateral sclerosis) neurodegenerative diseases [1]. Both of these two neurodegenerative diseases share some common characteristics. For example, a large proportion of ALS patients present behavioral and cognitive changes that are similar to the symptoms of FTLD [2], and part of the FTLD-U patients develop MND (motor neuron disease) syndromes [3]. The most significant overlap between FTLD-U and ALS patients is the insoluble ubiquitin inclusion pathology [1]. Although intensive studies have discussed about the pathology of FTLD-U and ALS diseases, it still remains unclear whether the ubiquitin inclusion in both FTLD-U and ALS is correlated with the aggregation of specific proteins or through a generalized defect in protein ubiquitination and degradation. TDP-43 was discovered as a major component within the insoluble ubiquitin inclusions in about 45–50% sporadic and familial FTLD-U as well as in 95–97% sporadic and familial ALS patients [4–8]. TDP-43-UBI (+) inclusions are commonly observed among frontal, temporal and parietal lobes, and hippocampus in FTLD-U patients’ brains, and the motor cortex, brainstem and spinal cord in ALS patients’ brains [6, 7]. TDP-43 is known to be ubiquitinated, hyperphosphorylated, cleaved to C-terminal fragments and redistributed from nucleus to cytoplasm during pathological progression [6, 7, 9, 10]. Under normal conditions, TDP-43 is important for RNA splicing and transport, while dysregulated TDP-43-mediated RNA splicing is observed in ALS patients [11, 12]. A wide spectrum of pathological processing-related events that contribute to TDP-43 aggregate formation have been reported, including mutations within the C-terminal glycine-rich region [13], hyperphosphorylation [14, 15], ubiquitination [6], nucleus/cytoplasm mislocalization [16], abnormal cleavage into 25/35 kDa C-terminal fragments [10, 17], changes in solubility [18], prolonged protein half-life [13], formation of detergent-insoluble cytosolic aggregates in the central nervous system [6, 7], and blockage of the ubiquitin proteasome system (UPS) or autophagy degradation pathways [19, 20]. Our previous work has demonstrated that TDP-43 can be degraded by the proteasome or autophagy, while mis-metabolism of TDP-43 results in pathological TDP-43 accumulation and aggregate formation [19]. Soluble TDP-43 is mostly degraded by the UPS, while excess insoluble aggregated TDP-43 is typically removed by autophagy [20]. However, in FTLD-U and ALS patients, the accumulation of TDP-43 within insoluble ubiquitin positive inclusions suggests that dysregulation of genes/factors involved in ubiquitination-related metabolism cascades likely contributes to TDP-43 proteinopathies. Although TDP-43 is the major constituent of ubiquitin positive inclusions within FTLD-U and ALS patient brains, the effect of TDP-43 ubiquitination on its pathological processing is not completely understood, and the E3 ligase that involves TDP-43-UBI (+) proteinopathies has not been identified yet.

It is well-known that numerous RING finger domain-containing proteins act as E3 ubiquitin ligases [21]. The RING finger proteins bind two zinc atoms in a cross-braced conformation via cysteine-rich C3HC4 or C3H2C3 RING finger domains [22, 23]. The unique amino acid sequence containing conserved Cys and His residues in the RING finger domain is **C**–X_2_–**C**–X_(9–39)_–**C**–X_(1–3)_–**H**–X_(2–3)_–**C/H**–X_2_–**C**–X_(4–48)_–**C**–X_2_–**C**, where X can be any amino acid. The classical RING finger sequence, RING-HC/C3HC4, has a histidine at the fourth coordinating position and a cysteine at the fifth [22, 23]. The brain specific RING finger protein, Znf179 (also called brain finger protein (BFP), or RING finger protein (RNF) 112**)**, is first identified in 1996 [24] which contains the classical C3HC4 sequence. Znf179 is predominantly expressed in brain regions, including cerebral cortex, hippocampus, lateral amygdaloidal nucleus, ventromedial hypothalamus, and cerebellum. Its expression gradually increases during embryogenesis in the developing brain and reaches the highest level in adult brain tissue. Znf179 is critical for neuronal differentiation in embryonic carcinoma P19 cells and cerebellar granule primary cultured cells [25], suggesting that it plays an important role during neuronal development. Microarray data (Gene Expression Omnibus; GEO) revealed that Znf179 is significantly downregulated in mouse models of neurodegenerative diseases such as Huntington’s disease (GEO: GDS717) and ALS (GEO: GSE10953) (Additional file 1: Figure S1), implying that Znf179 is associated with neurodegenerative pathology [26, 27]. However, the comprehensive cellular and molecular function of Znf179 is still largely unknown despite being discovered nearly two decades ago.

In this study, for the first time we provide clear evidence to demonstrate that Znf179 is a novel RING E3 ligase that plays an important role in regulating TDP-43-UBI (+)-related neurodegenerative pathologies. Znf179 possesses autoubiquitination activity that is dependent on the RING finger domain and is involved in modulating 26S proteasome activity. Using immunoprecipitation and mass spectrometry (MS), we found that the TDP-43 protein is a substrate of Znf179 E3 ligase. Both in vitro and in vivo ubiquitination assays showed that Znf179 can interact with and polyubiquitinate TDP-43 protein. Notably, the turnover rates and insoluble aggregates clearance of TDP-43 are regulated by Znf179 and the knockout of Znf179 in mice significantly increases the insoluble TDP-43 aggregates and punctate staining of TDP-43 inclusions in brain, suggesting that the E3 ligase, Znf179, is highly correlated with TDP-43-UBI (+) neuropathy. These findings support the notion that Znf179 could be a novel therapeutic target for combating TDP-43-UBI (+)-related neurodegenerative diseases, such as FTLD-U and ALS.

## Methods

### Cell culture, DNA transfection and Znf179 stable clone

N2a (Neuro-2a) cells were cultured in Eagle’s minimum essential medium, and 293 T cells were cultured in Dulbecco’s modified Eagle’s medium (Invitrogen, Carlsbad, CA), supplemented with 10% (*v*/v) fetal calf serum and 1% penicillin-streptomycin (Invitrogen). DNA transfection of N2a and 293 T cells was carried out with Lipofectamine 2000 (Invitrogen) according to the manufacturer’s instructions. To generate Znf179 stable clone, N2a cells were transfected with GFP-tagged Znf179 plasmids and then selected by G418 to generate the N2a GFP-mZnf179 stable cell line that stably expressed Znf179.

### Construction of mouse Znf179 gene targeting vector and generation of conventional Znf179 knockout mice

Animals used in this study were performed according to the guidelines of the Taipei Medical University Institutional Animal Care and Use Committee (IACUC) under permit number LAC-101-0048. All animals were housed in an air-conditioned vivarium with free access to food and water and a 12/12-h light/dark cycle. Technical services for mouse Znf179 gene targeting vector construction and conventional Znf179 knockout mice generation were provided by the Transgenic Mouse Model Core Facility of the National Core Facility Program for Biotechnology, National Science Council and the Gene Knockout Mouse Core Laboratory of National Taiwan University Center of Genomic Medicine [28].

Briefly, a 14.6-kb *Znf179* genomic DNA fragment coding for exons 1–15 were isolated from a BAC clone harboring 129/Sv genomic DNA (bMQ-194 N15 129S7AB2.2) (Geneservice, Cambridge, UK) and inserted into PL253 vector. The knockout strategy was to delete *Znf179* exons 4 to 7. The 5.6-kb BamHI-SepI fragment (short arm) and 7.6-kb EcoRI-NotI fragment (long arm) were rendered blunt, ligated to appropriate linkers, and inserted into the pKO-loxP targeting vector. The resultant targeting construct was then linearized by NotI digestion and was used for transfection of the R1 ES cell line by electroporation as previously described [29]. G418 (2 μM) and gancyclovir (10 μM) were used for transformant selection, and survived cell colonies were isolated and established as clones. These clones were genotyped by Southern blotting to ensure that homologous recombination had taken place. The ES cell clones were then transiently transfected with a vector expressing the Cre recombinase to delete the mouse *Znf179* exons 4 to 7 and the neo cassette. The correctly targeted ES clones were identified by PCR screening and then injected into C57BL/6 donor blastocysts to produce chimeras using a previously described technique [30]. After germ line transmission of the targeted mutation allele into heterozygous offspring of the chimera, the homozygous *Znf179* knockout mice were obtained by intercrossing the heterozygous *Znf179* knockout mice. To genotype the mice, genomic DNA was extracted from tail and analyzed by PCR. Thermal cycling was carried out for 40 cycles with denaturation at 94 °C for 30 s, annealing at 62 °C for 40 s, and extension at 72 °C for 1 min. The following primers were used to discriminate wild-type and mutant alleles: FP1, 5′-TGCTAATCTCTCCCTTGGTCCTC-3′, RP1–1, 5′-TTCCAGACAGATGGAGCAGG TG-3′, and RP1–2, 5′-TGCATCCCAGAACGCAAGTC-3′.

### Znf179-5A mutant plasmid construct

Site-directed mutants were generated using the plasmids pCMV-Tag2-Flag-mZnf179 and pGFP-mZnf179 (mouse Znf179) as templates. The critical Cys or His residues on the RING finger domain of Znf179 were mutated to Ala as the catalytically inactive Znf179-5A mutant. Several point mutations (C80A, C95A, H97A, C100A, C103A) were generated within the C3HC4 motif on the RING domain of Znf179 to create the Znf179-5A mutant. Site-directed mutagenesis (QuikChange kit; Stratagene, La Jolla, CA) was used to create sets of missense Znf179-5A mutations. The sequences of the mutagenized oligonucleotides were as follows: mZNF179-C80A, 5′- CCCGGGAGCCGCCCACCGCATCCATCTGTCTGGAAAG -3′; mZNF179-C95A, 5’-CCCATCTCGCTGGACGCAGGCCATGACTTCTGC -3′; mZNF179-H97A, 5′- CTCGCTGGACTGTGGCGCAGACTTCTGCATCCGATG -3′; mZNF179-C100A, 5′- GACTGTGGCCATGACTTCGCAATCCGATGCTTCAGCAC -3′; mZNF179-C103A, 5′- CATGACTTCTGCATCCGAGCATTCAGCACACACCGCATC -3′.

### In vitro ubiquitination assays

Immunoprecipitated Znf179 or TDP-43 proteins from 500 μg of whole cell lysate were mixed with ubiquitination reaction mixture including 10× ubiquitination buffer, 100 nM E1, 2.5 μM ubiquitin, 5 mM Mg^2+^-ATP and 2.5 μM His-tagged human E2 conjugating enzymes, and then incubated at 37 °C for 60 min. In the TDP-43 in vitro ubiquitination assay, immunoprecipitated endogenous TDP-43 from non-treated N2a cells were incubated with E1, ubiquitin, Mg^2+^-ATP, E2 enzyme UbcH5c. E3 ligase was derived from immunoprecipitated Flag-mZnf179 (from Flag-mZnf179-transfected 293 T cells) or GFP-mZnf179 (from N2a GFP-mZnf179 stable clone) pulled-down by anti-Znf179 antibody.

### 26S proteasome activity assay

Cultured 293 T cells were transfected with the GFP-fused ubiquitin mutant plasmid (Ub^G76V^-GFP) (#11941, Addgene, Cambridge, MA) in 6-well plates for 24 h, and the immunofluorescence of Ub^G76V^-GFP was measured with an ImageXpressXL fluorescence reader and quantified by MetaMorph software (Molecular Devices, Downingtown, PA). For quantitative analysis of Ub^G76V^-GFP fluorescence-positive cells, about 270 representative fields per well were taken, covering the entire well, and analyzed by MetaMorph software. The total number of DAPI-positive cells was counted and the GFP images were visually adjusted to determine a common threshold across all samples to eliminate background from Ub^G76V^-GFP fluorescence images. GFP signal was gated to exclude Ub^G76V^-GFP fluorescence-negative cells, and the images were then superimposed with corresponding DAPI images. The numbers of Ub^G76V^-GFP fluorescence-positive cells were calculated with the Integrated Morphometry Analysis tool in Metamorph. The percentages of Ub^G76V^-GFP fluorescence-positive cells were calculated according to the following formula: total number of GFP-positive cells / total number of cells. The relative ratio of the percentage of Ub^G76V^-GFP fluorescent positive cells were normalized to that of the Flag group (Relative ratio of the Flag group = 1).

### In gel digestion and mass spectrometry

To quantify the protein expression levels of 26S proteasome subunits in wild-type and Znf179-knockout mice brain, we used label-free *quantitative MS.* Hippocampus brain tissue dissected from wild-type and Znf179-knockout mice were lysed in lysis buffer (containing 50 mM Tris-HCl, pH 7.8, 150 mM NaCl, 0.1% Nonidet P-40 and protease inhibitor) and were run on SDS-PAGE (4–20% polyacrylamide gel) for 10 min to remove the NP-40 detergent. The gel was stained with Sypro Ruby (Invitrogen) and the bands including the whole hippocampus lysates were excised from gels. These gels were further cut into 1 mm^3^ cubes, washed twice with 50% acetonitrile in 25 mM ammonium bicarbonate and dried in a vacuum centrifuge. The gel pieces were then rehydrated in 10 μl 25 mM ammonium bicarbonate (pH 8.5) buffer containing 0.0225 μg trypsin for at least 16 h at 37 °C. The digested peptides were extracted using 50% acetonitrile and 5% formic acid, and dried. Samples were then re-dissolved in 1 μl 1% formic acid and 9 μl 50% acetonitrile / 0.1% formic acid. All samples to be analyzed were pre-cleaned and purified by a C18 ZipTip Column (Millipore, Temecula, California, USA) according to the manufacturer’s protocol. Eluted peptides were sequenced with a LTQ Orbitrap XL mass spectrometer (Thermo Fisher Scientific, MA, USA), and peptides were identified and analyzed with Mascot 2.3.02 (Matrix Science) software. The quantification of relative protein amounts in wild-type or Znf179-knockout mice brains was achieved by Porgenesis LC-MS (Nonlinear Dynamics, Durham NC, USA). The MS results were normalized against internal controls, BSA (bovine serum albumin) and β-actin. Potential networks and subcellular pathways were analyzed by Ingenuity Pathway Analysis (IPA). Identified proteins were further analyzed by Western blotting with specific antibodies: anti-PSMA1, anti-PSMC6 (Proteintech, Rosemont, IL, USA, Group Cat# 15839–1-AP RRID:AB_2237797), anti-PSMD10 (Proteintech Group Cat# 12342–2-AP RRID:AB_2172809).

To search for the candidate E3 ligase substrates or interacting proteins of Znf179, we used immunoprecipitation combined with mass spectrometer assays to analyze the Znf179-co-immunoprecipitated complex. Protein from whole brain lysates of wild-type and Znf179 knockout mice was immunoprecipitated with anti-Znf179 antibody and separated by SDS-PAGE (4–20% polyacrylamide gel). Protein bands corresponding to immuno-positive bands were excised from gels, in gel digested with trypsin and the peptides were pre-cleaned by C18 ZipTip Column (as described above). Eluted peptides were sequenced with a LTQ Orbitrap XL mass spectrometer (Thermo Fisher Scientific), and peptides were analyzed with Mascot 2.3.02 (Matrix Science) software. Potential networks and subcellular pathways were further analyzed by IPA (Additional file 2: Figure S3A).

### Ingenuity pathway analysis (IPA)

The online software package IPA provides computational algorithms to identify and dynamically generate significant biological networks and canonical pathways, and to further categorize specific physiological processes. It ranks networks by a score that takes into account the number of focus genes or protein domain and the size of the networks, indicating the likelihood of the focus genes or protein in a network being found together by chance. The higher the score (score = −log(*p*-value)), the lower is the probability of finding the observed Network Eligible Molecules in a given network by chance. IPA was further used to analyze the protein-protein interactions and protein networks that we are interested. The predicted protein interaction networks and canonical pathways were generated by IPA using inputs of gene identifiers.

### Immunoprecipitation

Immunoprecipitation was performed according to manufacturer’s description (Catch and Release IP Kit, Millipore). In brief, N2a cells stably expressing GFP-mZnf179 or 293 T cells transfected with 8 μg Flag-mZnf179 for 48 h were treated with 10 μM MG132 for 4 h. These cells were lysed in lysis buffer containing 50 mM Tris-HCl (pH 7.8), 150 mM NaCl, 0.1% Nonidet P-40, 0.5% Triton X-100 and protease inhibitor cocktail. For mouse brain lysates, wild-type and Znf179 knockout brain tissue was lysed in lysis buffer as described above. Five-hundred micrograms of total protein were mixed with 2 μl anti-Znf179 or anti-TDP-43 antibodies (Proteintech Group Cat# 12892–1-AP RRID:AB_2200505) and added to the Antibody Capture Affinity Ligand in 1× Wash Buffer to make a final volume of 500 ul. After incubation for 2 h at 4 °C, the immunocomplexes were gently washed three times with wash buffer followed by Elution Buffer to elute specific protein for further analysis by Western blotting with anti-Ubi (Enzo Life Sciences Cat# BML-PW0930 RRID:AB_10998070), anti-Znf179 and anti-TDP-43 antibodies.

### Cyclohexamide chase assay

N2a cells with or without stably expressing GFP-mZnf179 were transfected with Myc-hTDP-43 (human TDP-43) and then treated with cycloheximide (20 mg/ml) to inhibit further protein synthesis. The cells were harvested at the indicated time points. Cell lysates were immunoprecipitated by anti-Myc antibody (Millipore Cat# 05-724MG RRID:AB_568800) and the Myc-hTDP-43 protein levels were analyzed by Western blotting. The intensities of the TDP-43 protein bands were quantified, relative to the internal control of α-tubulin, using AlphaEaseFC software. The protein half-lives were fitted to the equation of the regression line, y = ax+b, obtained from each group where y is the ratio of relative protein levels and x is the time in hours (h).

### Immunohistochemistry and immunofluorescence

293 T cells grown on glass coverslips were transfected with plasmids as indicated. At 48 h post-transfection, the cells were fixed with 4% ice-cold paraformaldehyde at 4 °C for 20 min and then permeabilized with PBS with 0.5% Triton X-100 for 7 min at room temperature. After blocking with 10% donkey serum for 1 h at room temperature, the cells were incubated overnight at 4 °C with indicated antibodies. After washing, the cells were incubated at room temperature for 1.5 h with DAPI (1:500) plus fluorescent-conjugated secondary antibodies (1:500).

As for brain tissues, animals were deeply anaesthetized with sodium pentobarbital (50 mg/kg) and intracardially perfused with 4% paraformaldehyde in PBS, pH 7.4. Brains were carefully removed and postfixed in the same solution at 4 °C for overnight. Fixed tissues were immersed in 30% sucrose in PBS until they sank to the bottom of the sucrose solution, and then were frozen in OCT and cryosectioned into 20 μm sections. For immunohistochemistry and immunofluorescence staining, tissue sections were permeabilized with 0.2% Triton X-100 for 10 min and blocked for nonspecific binding by 2% normal goat serum (NGS) plus 2% BSA in PBS for 1 h at room temperature. Proteins of interest were detected by specific antibodies prepared in blocking buffer, and the slides were incubated for 18 h at 4 °C. After extensive wash with PBS, slides were incubated with the corresponding secondary antibodies for 30 min at RT. After washing by PBS three times, slides were mounted with anti-fade solution (Vector Laboratories, Burlingame, CA, USA). For immunohistochemistry, the DAB reagent (Vector) was used prior to counterstaining with hematoxylin and mounting. The images were examined on a CCD containing fluorescence microscope.

### Solubility and biochemical analysis

To examine the solubility profile of endogenous TDP-43 or exogenous Myc-hTDP-43, sequential extractions were performed. Transfected cells were washed twice with phosphate-buffered saline, lysed in cold RIPA buffer, and then sonicated. Cell lysates were cleared by centrifugation at 100,000×g for 30 min at 4 °C to generate the RIPA-soluble samples. To prevent carry-over, the pellets were washed twice (re-sonicated and re-centrifuged between washes) with RIPA buffer. RIPA-insoluble pellets were then extracted with urea buffer (7 M urea, 2 M thiourea, 4% CHAPS, 30 mM Tris-HCl, pH 8.5), sonicated, and centrifuged at 100,000×g for 30 min at room temperature. Protease inhibitors were added to all buffers prior to use (1 mM PMSF and a mixture of protease inhibitors). The expression patterns of TDP-43 proteins were then analyzed by Western blotting.

### Statistical analysis

All experiments were conducted at least in triplicate, and the results are expressed as the mean ± SEM. The statistical analyses were conducted using one-way analysis of variance (ANOVA) or Student’s *t*-test. The following *P* values were considered significant: *, *P* < 0.05; **, *P* < 0.01; ***, *P* < 0.001.

## Results

### Znf179 possesses E3 ubiquitin ligase activity

**Fig. 1:**
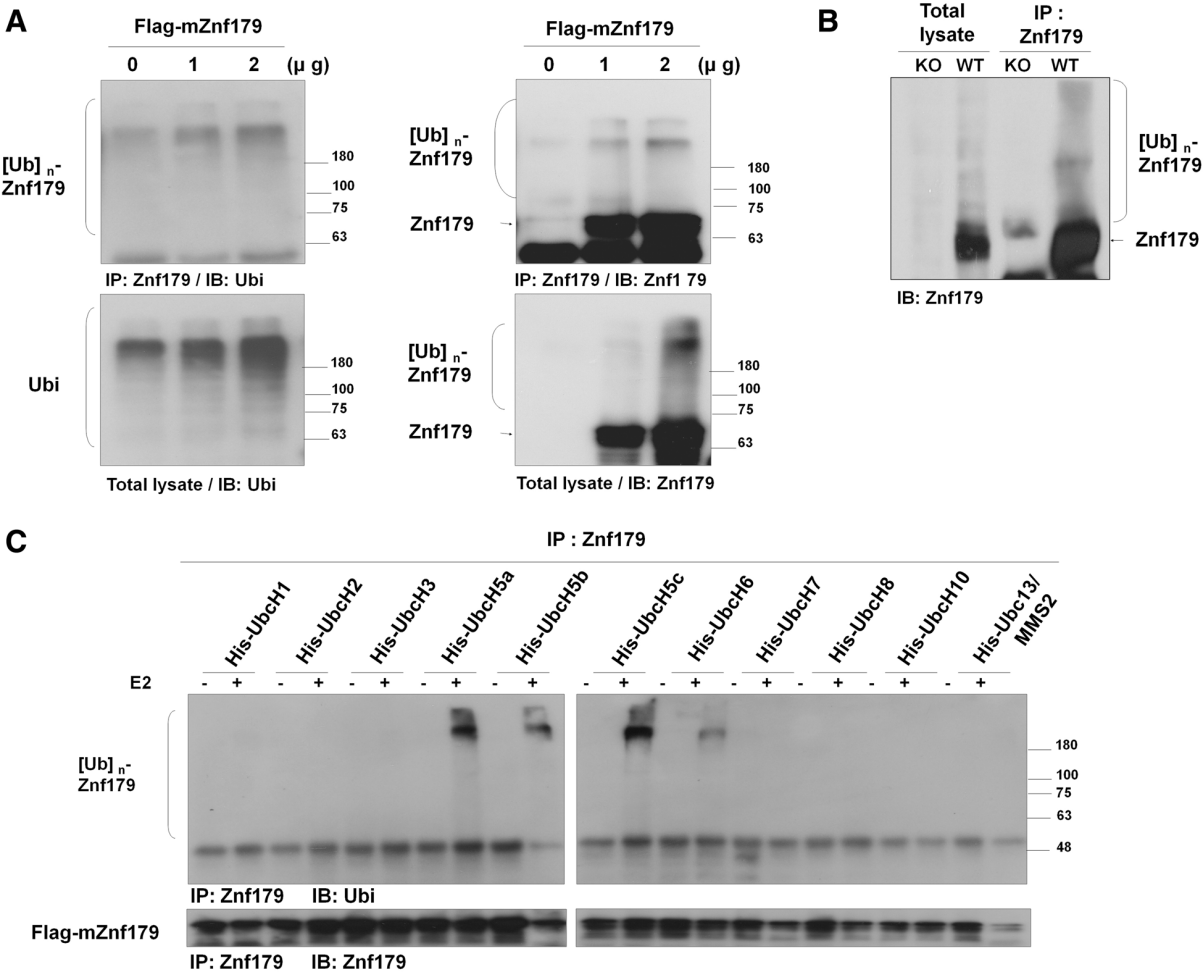
Autoubiquitination of Znf179 in the presence of E2 conjugating enzyme UbcH5 family proteins in vitro. **a** 293 T cells transfected with different doses (0, 1, 2 μg) of Flag-mZnf179 for 48 h were immunoprecipitated with anti-Znf179 antibody and analyzed by Western blotting with anti-Ubi and anti-Znf179 antibodies. **b** Total brain lysates from wild-type or Znf179-knockout mice were immunoprecipitated and analyzed by Western blotting with an anti-Znf179 antibody. **c** 293 T cells were transfected with Flag-mZnf179 for 48 h. The immunoprecipitated Flag-mZnf179 proteins were introduced to the mixture of purified E1, ubiquitin, Mg^2+^-ATP and one of several purified His-E2 enzymes to perform in vitro ubiquitination assays. The ubiquitination patterns of Znf179 were detected by Western blotting with anti-ubiquitin and anti-Znf179 antibodies

Numerous proteins containing RING finger domains act as E3 ubiquitin ligases [21, 22], and autoubiquitination is a notable feature for RING E3 ubiquitin ligases, resulting in proteasome degradation or functional alteration of the E3 ligase protein itself [31]. The enzymatic activity of RING E3 ligases can thus be monitored through probing autoubiquitination modifications [21, 32]. Znf179 contains the classical C3HC4 RING finger domain, prompting us to investigate its role as a RING-type E3 ubiquitin ligase. To test this hypothesis, we determined whether Znf179 has autoubiquitination activity. We first identified the ubiquitination patterns of Znf179 in cultured cells or mouse brain tissue. We transfected 293 T cells with different amounts of Flag-tagged mZnf179 plasmid (0, 1, 2 μg) for 48 h and Flag-mZnf179 proteins were immunoprecipitated with anti-Znf179 antibodies. Endogenous Znf179 protein was undetectable under normal conditions in 293 T cells (lower right panel in Fig. 1a). The polyubiquitination pattern of Znf179 was observed in a dose-dependent increase (upper two panels and lower right panels in Fig. 1a). Furthermore, the polyubiquitination level of endogenous proteins was also increased in lysates from Znf179 overexpressing cells (lower left panel in Fig. 1a), suggesting that Znf179 possesses E3 ubiquitin ligase activity which mediates the ubiquitination of numerous endogenous proteins. In addition, we also examined the expression and polyubiquitination of Znf179 in wild-type and Znf179 knockout mouse brains. We observed strong signal for polyubiquitinated Znf179 in brain lysates from wild-type mice compared to those of Znf179-knockout mice (Fig. 1b). These results thus demonstrate that Znf179 is an E3 ligase that mediates polyubiquitination in Znf179-overexpressing cells and in mouse brains.

**Fig. 2:**
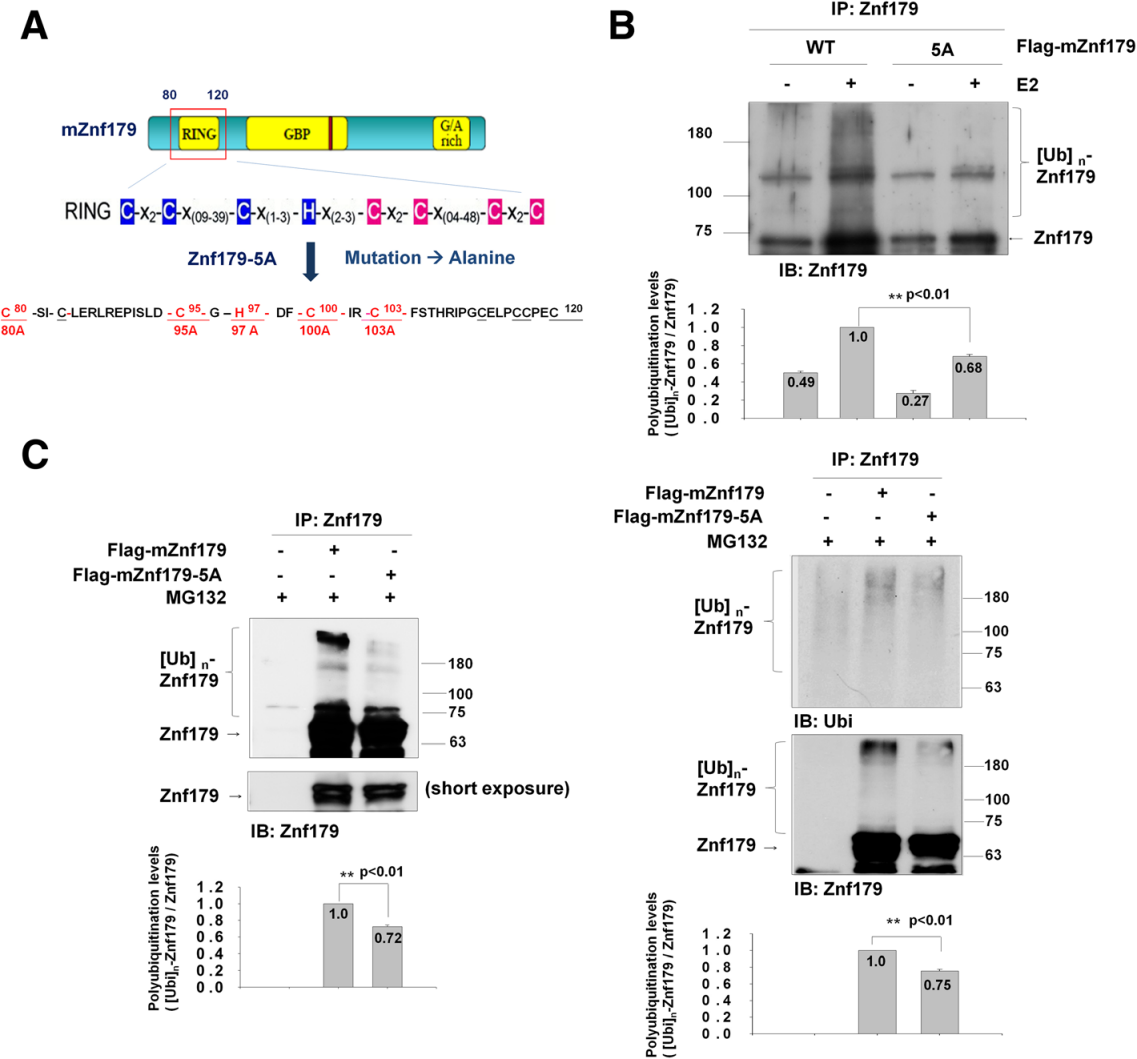
Autoubiquitination of Znf179-5A mutant is decreased in vitro and in vivo. **a** A schematic diagram of the Znf179-5A mutants is shown. Several point mutations (C80A, C95A, H97A, C100A, C103A) were generated within the C3HC4 motif on the RING domain of Znf179 to create the Znf179-5A mutant. **b** 293 T cells were transfected with Flag-mZnf179 or Flag-mZnf179-5A mutant for 48 h. The immunoprecipitated Flag-mZnf179 or Flag-mZnf179-5A proteins were introduced to the mixture of purified E1, ubiquitin, Mg^2+^-ATP and purified His-UbcH5c E2 enzymes to perform in vitro ubiquitination assays. The ubiquitination patterns of Znf179 and its immunoprecipitation levels were detected in parallel by Western blotting with anti-ubiquitin and anti-Znf179 antibodies. **c** and **d** Cell lysates from N2a cells (**c**); or 293 T cells (**d**) expressing Flag-mZnf179 and Flag-mZnf179-5A mutant were immunoprecipitated with anti-Znf179 antibody and analyzed with anti-ubiquitin and anti-Znf179 antibodies by Western blotting. **b**-**d** Data were presented as the mean ± SEM (*006E* = 3) of at least three independent experiments (** *p* < 0.01, groups were compared by t-test, two-tailed p values). The polyubiquitination levels are compared between [Ubi]n-Flag-mZnf179/Flag-mZnf179 and [Ubi]n-Flag-mZnf179-5A/Flag-mZnf179-5A

To further confirm the E3 ligase autoubiquitination activity of Znf179, and to screen for potential E2 conjugating enzymes, we performed a series of in vitro ubiquitin ligase assays. Flag-mZnf179 proteins were immunoprecipitated from transfected 293 T or N2a cell lysates with anti-Znf179 antibody and incubated with purified E1 enzyme, ubiquitin, Mg^2+^-ATP and purified E2 conjugating enzymes. The autoubiquitination patterns of Znf179 were analyzed with anti-ubiquitin and anti-Znf179 antibodies by Western blotting (Fig. 1c). In the presence of UbcH5 family E2 conjugating enzymes, including UbcH5a, UbdH5b, UbcH5c and UbcH6, autoubiquitination of Znf179 was observed (Fig. 1c). These results demonstrate that Znf179 is an E3 ubiquitin ligase which is able to autoubiquitinate itself in cooperation with UbcH5 family E2 conjugating enzymes. E3 ligase activity dependents on the integrity of the RING finger domain, and mutations in the essential zinc-correlated Cys or His residues within the RING finger domain are known to eliminate RING-E2 interactions and E3 ligase activity [32–36]. To determine whether the RING finger domain of Znf179 is essential for its E3 ligase activity, the critical Cys or His residues on the RING finger domain of Znf179 were mutated to Ala as the catalytically inactive Znf179-5A mutant (Fig. 2a). Immunoprecipitated wild-type Flag-mZnf179 or mutant Flag-mZnf179-5A from 293 T or N2a cells were incubated with purified E1, ubiquitin, Mg^2+^-ATP and E2 conjugating enzyme (UbcH5c). The autoubiquitination signal of the Znf179-5A mutant was diminished compared to that of wild-type Znf179 in the presence of UbcH5c in vitro (Fig. 2b). To further confirm the in vitro assay results, we also overexpressed wild-type Flag-mZnf179 or mutant Flag-mZnf179-5A in N2a or 293 T cells and immunoprecipitated each of the proteins with anti-Znf179 antibody. The polyubiquitination level of Flag-mZnf179-5A mutant was obviously less than that of wild-type Flag-mZnf179 in N2a cells (Fig. 2c) or 293 T cells (Fig. 2d). In combine, the in vitro and in vivo results strongly suggest that Znf179 is a RING E3 ligase per se, and the Cys and His residues on the RING finger domain are critical for the Znf179 E3 ligase catalytic activity which regulates its own autoubiquitination capability.

### Znf179 modulates proteasome activity

**Fig. 3:**
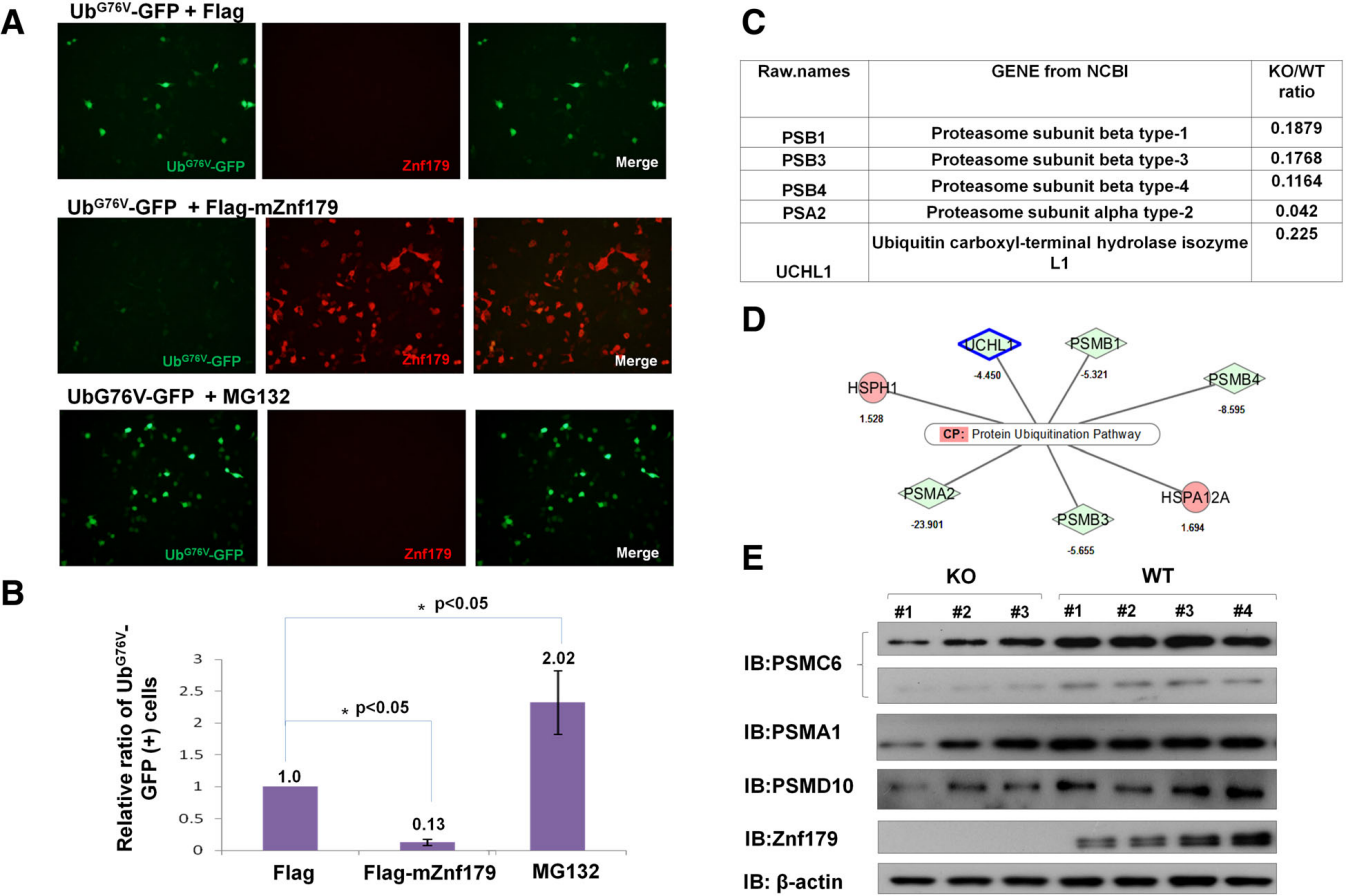
Znf179 enhances 26S proteasome activity by regulating the protein expression levels of 19S and 20S proteasome subunits. **a** 293 T cells were co-transfected with Flag-tagged mZnf179 and Ub^G76V^-GFP for 24 h and treated with 10 μM MG132 for 6 h as positive control. The cells were stained with anti-Znf179 antibody (red) and the fluorescence intensity of Ub^G76V^-GFP (green) was quantified by the ImageXpress® XL System. **b** The quantified data showed the fluorescence intensities of Ub^G76V^-GFP-positive cells in Flag-mZnf179-transfected cells or MG132-treated cells. Data were presented as Mean ± SEM of at least three independent experiments (* *p* < 0.05, groups were compared by *t*-test*,* two-tailed *p* values). **c** and **d** The protein expression levels of 26S proteasome subunits in hippocampus lysates from wild-type and Znf179 knockout mice were quantified by mass spectrometry analysis. After In-gel trypsin digestion and Zip-tip purification, samples were run on a LTQ-Orbitrap mass spectrometer and analyzed by Mascot 2.3.02 software to identify matched proteins. Further quantification was accomplished with Porgenesis LC-MS software. The differential expressions of peptides related to the 26S proteasome were presented as the ratio in knockout to wild-type. The MS results were normalized by the internal controls, BSA and β-actin, and were further classified by Ingenuity pathway analysis (IPA). Several proteins with significant differences (expression level ≥ 1.3-fold, *p* < 0.05, peptides ≥2) were associated with ubiquitin-proteasome systems, including the 20S proteasome subunits PSMA2, PSMB1, PSMB3 and PSMB4. **e** Protein expression patterns of the 19S proteasome subunits, PSMC6 and PSMD10, and 20S proteasome subunits, PSMA1 between wild-type and Znf179-knockout mice brain were analyzed by Western blotting. β-actin was used as an internal control

Numerous E3 ubiquitin ligases are involved in regulation of proteasome activity, such as Parkin activates the 26S proteasome through interacting with 19S proteasome subunits and participating in the assembling and maintenance of the 26S proteasome [37]. To investigate whether the RING E3 ligase, Znf179, plays a role in regulating the ubiquitin proteasome activity, we used a proteasome specific substrate, a GFP-fused ubiquitin mutant protein (Ub^G76V-^GFP). Mutation of Gly76 to Val on ubiquitin prevents its removal by deubiquitination machinery, leading to continuous polyubiquitination of GFP, which acts as a signal to direct the protein toward 26S proteasomal degradation [38]. Indeed, the percentage of Ub^G76V^-GFP fluorescence positive (+) cells is inversely proportional to the intracellular activity of the 26S proteasome. Accordingly, the relative ratio of Ub^G76V^-GFP (+) cells was increased in the presence of the proteasome inhibitor, MG132, comparing to that of Flag control group (Fig. 3a and b). In contrast, the relative ratio of Ub^G76V^-GFP (+) cells was significantly lower in cells expressing Flag-mZnf179 than that of controls group expressing Flag only (Fig. 3a and b). These results thus suggest that overexpression of Znf179 accelerates the degradation rate of Ub^G76V^-GFP through enhancing proteasome activity.

To explore the mechanism of Znf179 regulated proteasome activity, the protein expression levels of 26S proteasome subunits from hippocampus tissue lysates of both wild-type and Znf179-knockout mice were analyzed by label-free quantitative MS. We then processed the MS results using IPA and showed that several 20S proteasome subunits (PSMA2, PSMB1, PSMB3 and PSMB4) and ubiquitin-proteasome system-related proteins (UCHL1, HSPA12A and HSPH1) were decreased in Znf179-knockout mice compared to those in wild-type mice (Fig. 3c and d). We next analyzed the protein expression levels of several other 19S and 20S proteasome subunits by Western blotting and found that the 19S proteasome subunits, PSMC6 and PSMD10, and 20S proteasome subunit, PSMA1, were decreased in Znf179-knockout mice (Fig. 3e). Taken together, these findings suggested that overexpression of Znf179 increases the activity of 26S proteasome whereas knockdown of Znf179 decreases the protein expression levels of 26S proteasome subunits. Our results provided an important notion that Znf179 was an E3 ligase involving in regulating 26S proteasome activity through modulating the protein levels of 19S or 20S proteasome subunits.

### Znf179 interacts with and mediates the polyubiquitination of TDP-43

**Fig. 4:**
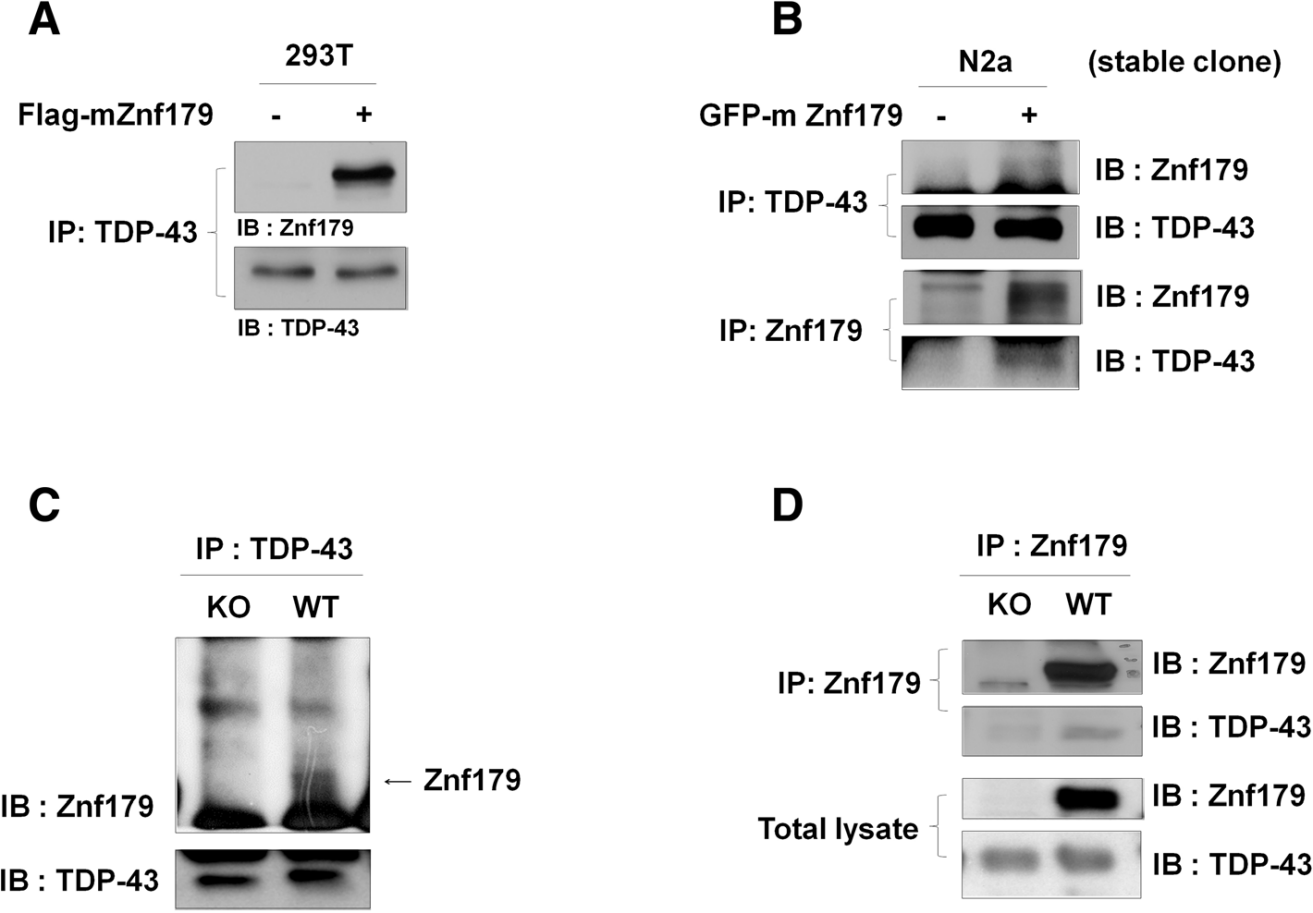
Znf179 interacts with TDP-43. **a** and **b** 293 T cells transiently transfected with Flag-mZnf179 (**a**) or N2a cells stably expressing GFP-mZnf179 (**b**) were immunoprecipitated with anti-TDP-43 or anti-Znf179 antibodies and immunoblotted with ant-TDP-43 and anti-Znf179 antibodies. **c** and **d** The total brain lysates of wild-type or Znf179-knockout mice were immunoprecipitated with either anti-TDP-43 or anti-Znf179 antibodies and immunoblotted with both anti-Znf179 and anti-TDP-43 antibodies

After identifying Znf179 as a RING E3 ubiquitin ligase possessing autoubiquitination feature and regulating proteasome activity, we further searched for its candidate E3 ligase substrates or interacting proteins by LC-MS. We immunoprecipitated Znf179 from the whole brain lysates of wild-type and Znf179-knockout mice with anti-Znf179 antibodies and then probed the identity of the pulled-down proteins with a LTQ-Orbitrap XL mass spectrometer. MS data was analyzed by Mascot software (Additional file 2: Figure S3A). Our results revealed that FTLD-U and ALS-related neuropathological TDP-43 protein is one of the substrates of Znf179 E3 ligase. To validate these results, we first used further immunoprecipitation assay to test whether Znf179 interacts with TDP-43 to form a Znf179/TDP-43 complex. Lysates of 293 T cells being transiently transfected with Flag-mZnf179 (Fig. 4a) or N2a cells stably expressing GFP-mZnf179 (Fig. 4b) were immunoprecipitated with anti-TDP-43 or anti-Znf179 antibodies and immunoblotted with anti-TDP-43 and anti-Znf179. We found that endogenous TDP-43 co-immunoprecipitated with either Flag-mZnf179 or GFP-mZnf179 (Fig. 4a and b). Moreover, the interaction between endogenous Znf179 and TDP-43 was also observed in wild-type mouse brain lysates (Fig. 4c and d), confirming that Znf179 interacts with TDP-43 in both cultured cells and mouse brain.

**Fig. 5:**
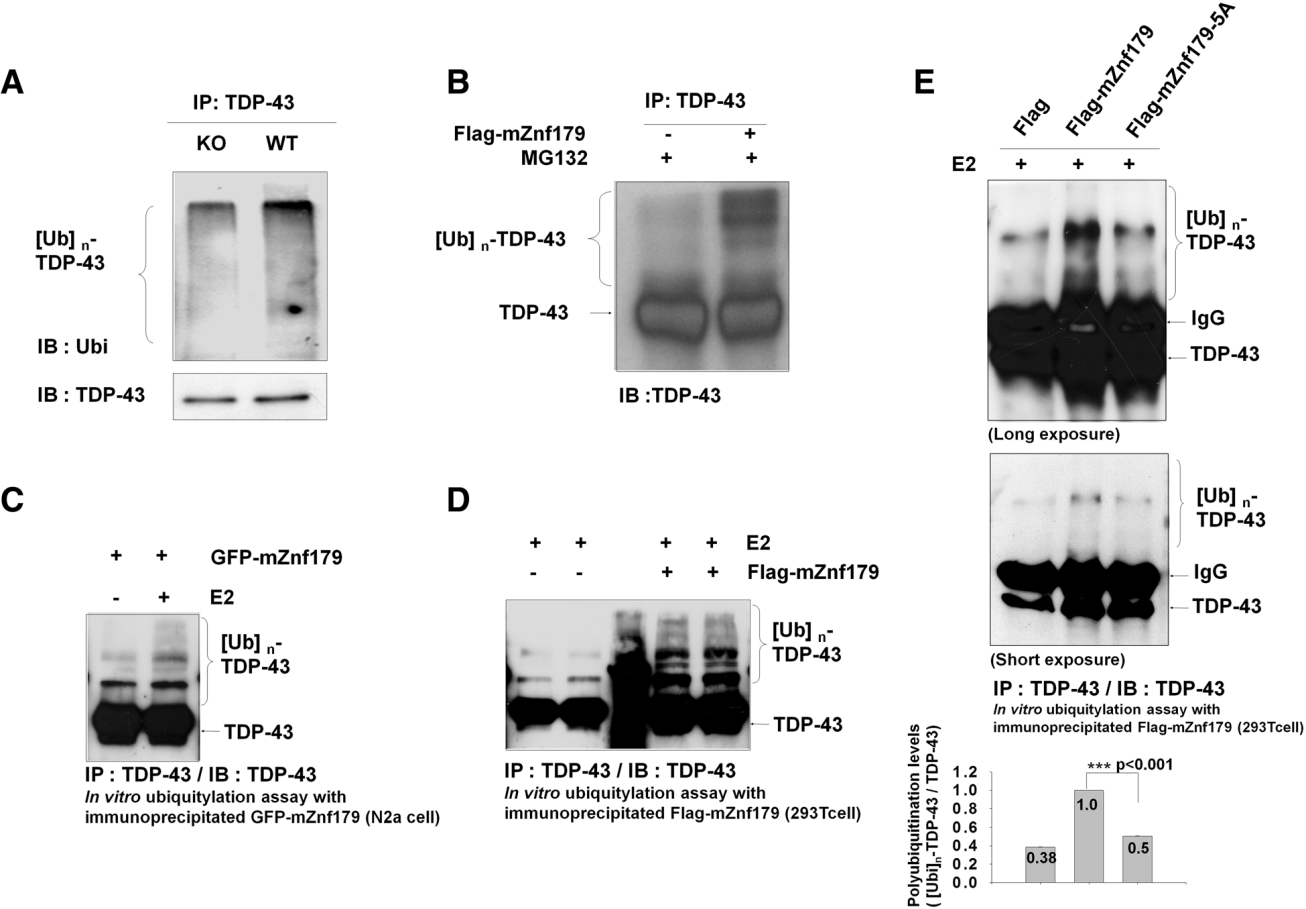
Znf179-mediates TDP-43 polyubiquitination in vitro and in vivo*.* **a** and **b** The total brain lysates of wild-type or Znf179-knockout mice (**a)** or 293 T cells transfected with Flag-mZnf179 and treated with 10 μM MG132 for 4 h (**b**) were immunoprecipitated by anti-TDP-43 antibody and analyzed by Western blotting with anti-ubiquitin or anti-TDP-43 antibodies. **c**, **d** and **e** For in vitro polyubiquitination assays, endogenous TDP-43 that was immunoprecipitated with anti-TDP-43 antibody from non-treated N2a cells lysates was included in a mixture of purified E1, ubiquitin, Mg^2+^-ATP, UbcH5c E2 conjugating enzyme, and the E3 ligase, Znf179. The Znf179 E3 ligase was immunoprecipitated from N2a cells stably expressing GFP-mZnf179 (**c**) or from 293 T cells transiently transfected with Flag-mZnf179 or with Flag-mZnf179-5A mutant (**d** and **e**). The ubiquitination levels of TDP-43 were analyzed by anti-TDP-43 antibody. Data were presented as the mean ± SEM (*n* = 3) (*** *p* < 0.001, groups were compared by *t*-test*,* two-tailed *p* values). The polyubiquitination levels of TDP-43 ([Ubi]n-TDP-43 / TDP-43) were compared between Flag-mZnf179 and Flag-mZnf179-5A groups (**e**)

We then tested whether Znf179 mediates the polyubiquitination of TDP-43. First, we found that polyubiquitinated TDP-43 was abundant in wild-type mouse brain compared to that in Znf179 knockout mouse brain (Fig. 5a). We further evaluated the polyubiquitination patterns of endogenous TDP-43 in 293 T cells transfected with Flag-mZnf179 in the presence of the proteasome inhibitor, MG132, and found that TDP-43 was polyubiquitinated in 293 T cell overexpressing Flag-mZnf179 (Fig. 5b). To further support the notion that Znf179 directly mediates TDP-43 polyubiquitination, we carried out the in vitro ubiquitination assays using TDP-43 immunoprecipitates as substrate (from N2a cells), along with immunoprecipitated Flag-mZnf179 or GFP-mZnf179 (from Flag-mZnf179 transfected 293 T cells or N2a cells stably expressing GFP-mZnf179) as an E3 ligase in the presence of purified UbcH5c, an E2 ubiquitin-conjugating enzyme. Enhanced polyubiquitination of immunoprecipitated TDP-43 was indeed observed in the presence of Flag-mZnf179 or GFP-mZnf179 along with UbcH5c E2 conjugating enzyme (Fig. 5d and d). To further confirm that the RING finger domain of Znf179 is critical for its E3 ligase activity to mediate TDP-43 polyubiquitination, we compared the polyubiquitination levels of TDP-43 in the presence of wild-type Znf179 or the catalytically inactive Znf179-5A mutant along with purified UbcH5c. The polyubiquitination level of immunoprecipitated TDP-43 was significantly decreased in the presence of Znf179-5A, compared to wild-type Znf179 (Fig. 5e). In combined, these data indicate that Znf179 is an E3 ubiquitin ligase for TDP-43, and the RING finger domain is critical for Znf179-mediated TDP-43 polyubiquitination.

### Znf179-mediated polyubiquitination of TDP-43 accelerates degradation and attenuates aggregate formation

**Fig. 6:**
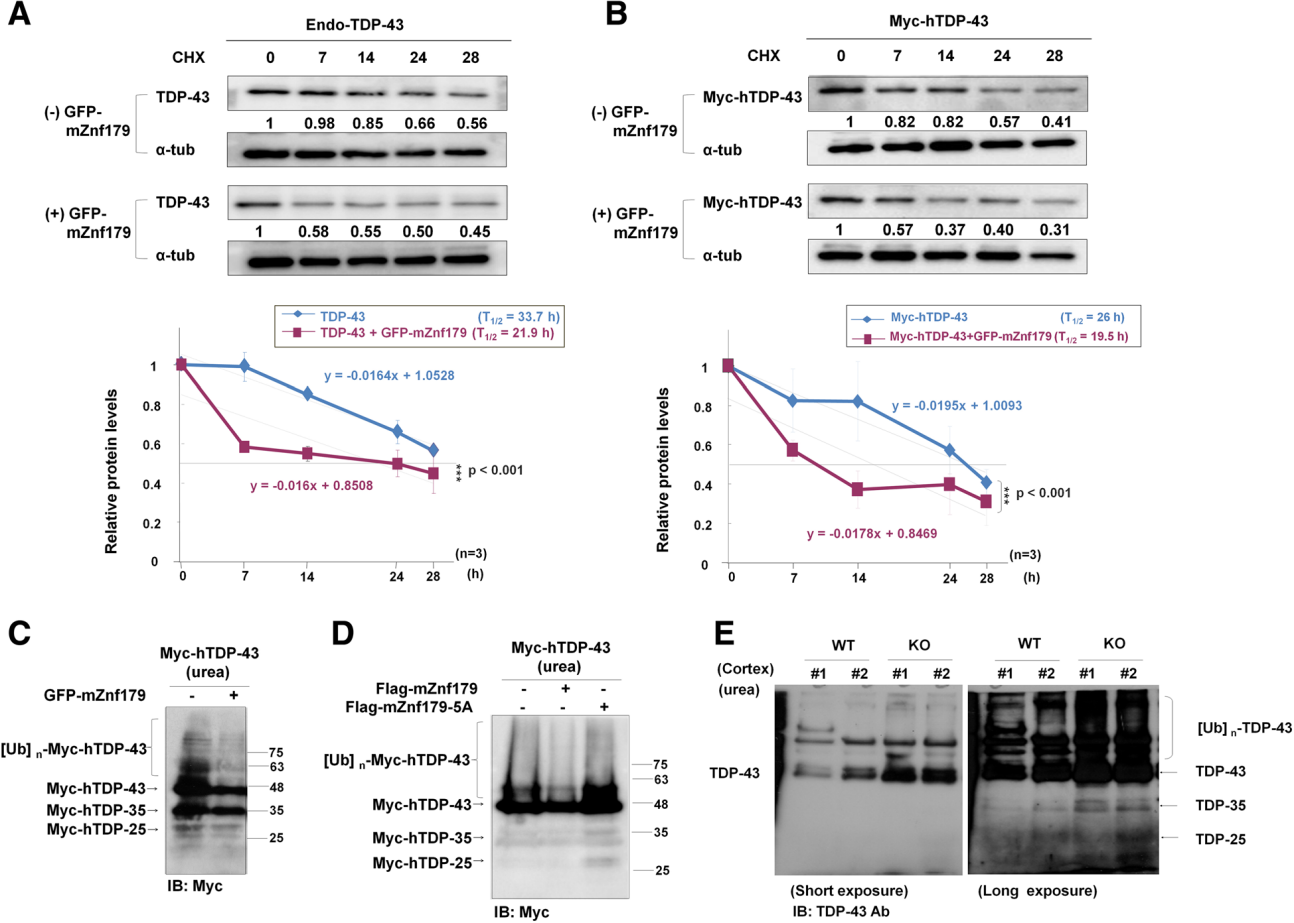
Znf179 enhances the degradation rate of TDP-43 protein and alters the solubility of TDP-43. **a** and **b** Endogenous TDP-43 (**a**) or Myc-hTDP-43 (**b**) in N2a cells with or without stably expressed GFP-mZnf179 was treated with cycloheximide (20 mg/ml) for different time periods. The protein levels of the Myc-hTDP-43 were analyzed by western blotting with anti-TDP-43 or anti-Myc antibody. The graph below the blots showed a quantification of the relative values at each time point, from which the half-lives of the proteins were estimated. Half-life was calculated by linear regression. Data were presented as the mean ± SEM (*n* = 3) (*** *p* < 0.001, groups were compared by *t*-test*,* two-tailed *p* values). **c** N2a cells with or without stably expressing GFP-mZnf179 were transiently transfected with Myc-hTDP-43 for 48 h, and analyzed by western blotting with anti-Myc antibody. **d** N2a cells transiently transfected with Myc-hTDP-43 and wild-type Flag-mZnf179 or Flag-mZnf179-5A mutant for 48 h were further analyzed by western blotting with anti-Myc antibody. **e** The cortex from wild-type and Znf179 knockout brains at 4 months old was extracted by urea buffer to probe the insoluble TDP-43 fraction and analyzed by immunoblotting with anti-TDP-43 antibody

Under normal condition, ubiquitinated TDP-43 is majorly degraded by proteasome or autophagy degradation pathways, and mis-metabolism of TDP-43 would result in pathological TDP-43 accumulation and TDP-43 aggregates formation [19]. Up to this point, we had shown that E3 ligase Znf179 not only enhances 26S proteasome activity (Fig. 3), but also mediates the polyubiquitination modification of TDP-43 protein in vivo and in vitro (Fig. 5). Based on these findings, we proposed that Znf179-enhanced proteasome activation may accelerate the clearance of TDP-43 protein and modulate the steady-state turnover rate of TDP-43 protein. To analyze the impact of Znf179-mediated TDP-43 polyubiquitination on TDP-43 metabolism, we first evaluated the degradation rates of both endogenous TDP-43 and exogenous overexpressed Myc-tagged hTDP-43 protein in the presence or absence of stably expressed GFP-mZnf179 in N2a cells. Cycloheximide chase experiments showed that the stable expression of GFP-mZnf179 decreased the half-life of endogenous TDP-43 from 33.7 h to 21.9 h (Fig. 6a). We then transfected Myc-tagged hTDP-43 in N2a cell with or without stably expressed GFP-mZnf179 for 48 h, and then treated these transfected cells with cycloheximide for indicated time periods. The stable expression of GFP-mZnf179 also decreased the half-life of the overexpressed full-length Myc–hTDP-43 from 26 h to 19.5 h (Fig. 6b). The effect of Znf179 on the TDP-43 half-life thus indicates that Znf179-mediated TDP-43 polyubiquitination accelerates the degradation of both endogenous and exogenous TDP-43 to maintain the steady-state turnover rate of TDP-43 protein.

In FTLD/ALS patients, the expression of TDP-43 is around 1.5 fold higher than that in healthy individuals [39, 40], which could be resulted from mis-metabolism of TDP-43 proteins [19]. The excess amount of TDP-43 in FTLD/ALS patients’ brain has a propensity to form insoluble TDP-43 deposits, since TDP-43 proteins is intrinsically an aggregation prone protein [41], which can be observed in the form of polyubiquitinated and truncated 35 kDa/25 kDa TDP-43 fragments in urea fraction [6, 13]. Up to this point, we have shown that Znf179-mediated TDP-43 polyubiquitination accelerates the degradation rates of TDP-43 proteins. We thus proposed that Znf179-mediated acceleration of TDP-43 turnover rates ameliorates the accumulation of insoluble deposits from excess amount of TDP-43 protein. To analyze the impact of Znf179 on insoluble TDP-43 aggregates formation, we first evaluate the insoluble deposits of overexpressed TDP-43 protein in the presence or absence of Znf179 in N2a cells. In N2a cells, the overexpression of exogenous Myc-hTDP-43 led to the formation of insoluble TDP-43 deposits, including polyubiquitinated full-length TDP-43 and truncated 35 kDa/25 kDa TDP-43 fragments (Fig. 6c and d, [19]), while insoluble polyubiquitinated full-length and truncated Myc-hTDP-43 deposition were indeed decreased in presence of GFP-mZnf179 or Flag-mZnf179 (Fig. 6c and d). These results thus suggest that Znf179-mediated polyubiquitination of TDP-43 accelerates the clearance of excess Myc-hTDP-43 protein and thereby decreases the insoluble Myc-hTDP-43 aggregates. This concept was further supported by the observation that insoluble Myc-hTDP-43 species were not diminished in Flag-mZnf179-5A mutant-expressing N2a cells (Fig. 6d), implying that the E3 ligase activity of Znf179 is a key factor to prevent the formation of pathological TDP-43 aggregates.

To further explore this notion, we compared the levels of insoluble polyubiquitinated full-length and truncated 35 kDa/25 kDa TDP-43 fragments from the cerebral cortex of wild-type and Znf179 knockout mice. Here we extracted the cortex and hippocampus of 4 month old wild-type and Znf179 knockout mice with urea buffer, and analyzed the levels of insoluble TDP-43 fractions, including polyubiquitinated full-length TDP-43 and truncated 35 kDa/25 kDa TDP-43 fragments [42], by immunoblotting with anti-TDP-43 antibody. We found that the expression levels of insoluble polyubiquitinated full-length TDP-43, and the truncated 35 kDa/25 kDa fragments were increased in the urea-soluble fraction from Znf179 knockout mice cortex at 4 months of age when comparing with the wild-type control (Fig. 6e). These data thus suggested that Znf179 deficiency results in reduced TDP-43 degradation rates, leading to increased accumulation of insoluble TDP-43 in cortex and hippocampus. Overall, our results demonstrated that Znf179-mediated TDP-43 polyubiquitination not only regulates the normal degradation of soluble TDP-43 through the UPS, but also attenuates the accumulation of insoluble TDP-43 deposits.

### Znf179 deficiency causes insoluble TDP-43 accumulation and TDP-43 aggregate formation

**Fig. 7:**
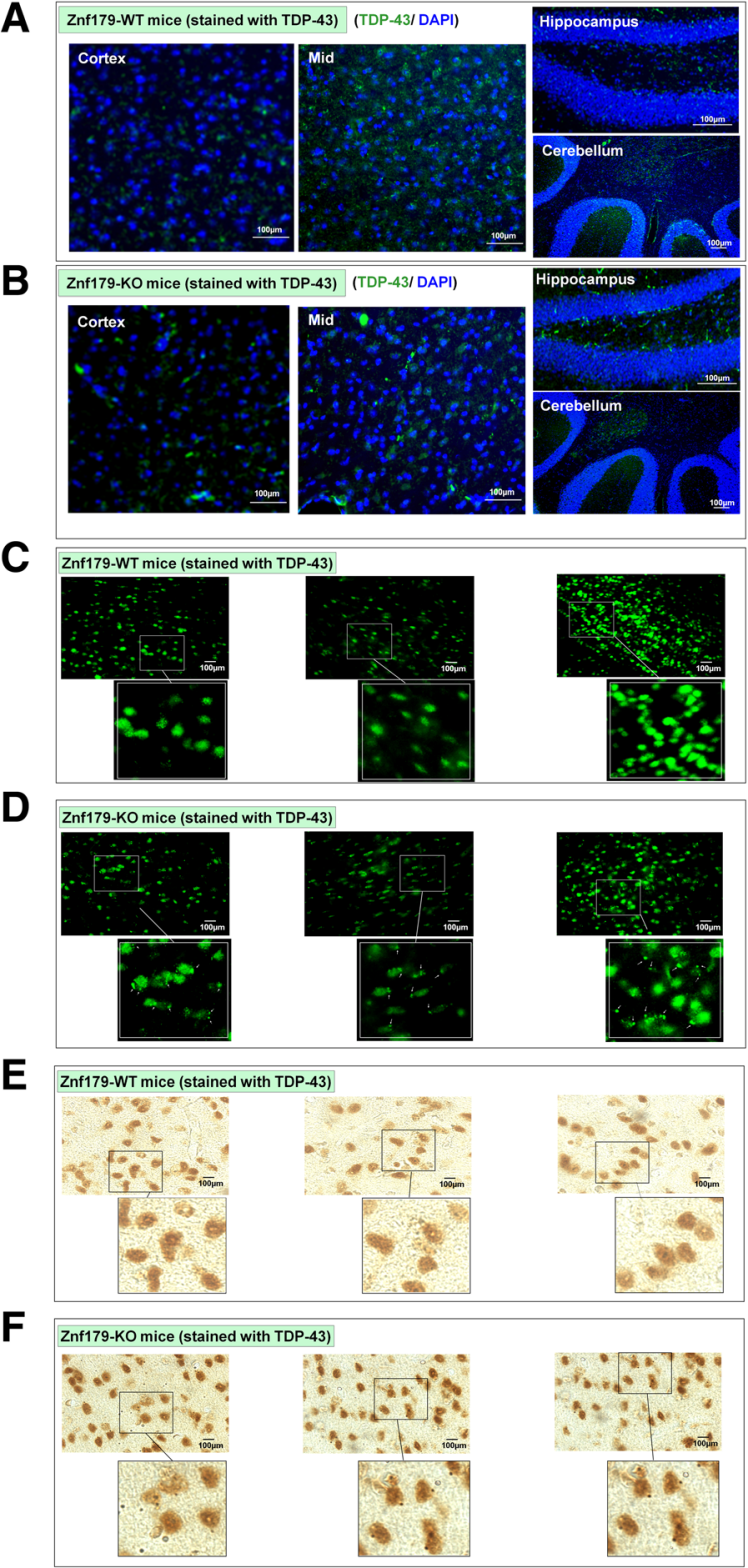
Knockout of Znf179 enhances TDP-43 aggregate formation in mice cortex and hippocampus. **a** and **b** The brain sections of wild-type (**a**) or Znf179-knockout mice (**b**) were stained with anti–TDP-43 antibody (green) and the nuclei were labeled with DAPI (blue). Scale bars = 100 μm. **c** and **d** The brain sections of wild-type (**c**) or Znf179-knockout mice (**d**) were stained with anti-TDP-43 antibody and the punctate staining of TDP-43 aggregates in the cortex region were indicated by white arrows. Scale bars = 100 μm. **e** and **f** The brain sections of wild-type (**e**) or Znf179-knockout mice (**f**) were stained with anti-TDP-43 antibody. Scale bars = 100 μm

The pathological TDP-43 protein, found in cytosolic insoluble inclusions, is known to be ubiquitinated in ALS and FTLD-U patients [6]. Mislocalization of TDP-43 from nucleus to cytosol is accompanied by reduced protein solubility and increased formation of protein aggregates. After finding that Znf179 is a novel E3 ubiquitin ligase, which mediates the polyubiquitination of TDP-43 protein and affects TDP-43 proteinopathies, we next tested the effect of Znf179 on TDP-43 aggregates formation in an animal model. In brains of wild-type and Znf179 knockout mice, we found that TDP-43 aggregates appeared as punctate staining in cortex, hippocampus and midbrain regions of Znf179-knockout mice (Fig. 7b), but not in wild-type control (Fig. 7a). On the other hand, TDP-43 aggregates are not observed in the cerebellum brain region within both wild-type and Znf179-knockout mice. Moreover, in the cortex region of Znf179-knockout mice brain, TDP-43 aggregates were mostly detected in the cytoplasm (Fig. 7d and f), but not in wild-type controls (Fig. 7c and e). These results thus suggested that Znf179 deficiency results in cytosolic accumulation to form cytosolic TDP-43 aggregates in mouse cortex, hippocampus and midbrain brain regions. In summary, our data indicated that Znf179-mediated TDP-43 polyubiquitination affects its cytosolic aggregates formation, suggesting that the Znf179-mediated polyubiquitination of TDP-43 is a key factor to determine the formation of pathological TDP-43 proteins.

## Discussion

In ALS and FTLD-U patients, TDP-43 has been identified as the major component of ubiquitin positive inclusions. Here, we identified Znf179 as an E3 ubiquitin ligase for TDP-43 that also exhibits autoubiquitination activity. Immunoprecipitation and MS analysis revealed that TDP-43 is a substrate of Znf179 E3 ligase. Both our in vitro and in vivo experiments confirmed that Znf179 interacts with and mediates polyubiquitination of TDP-43. In addition, Znf179 also regulates the proteasome activity by modulating the protein expression levels of 19S/20S proteasome subunits. In Znf179 overexpressing cells, polyubiquitination of TDP-43 not only accelerates degradation of TDP-43 protein through the UPS, but also enhances the removal of insoluble TDP-43 aggregates, implicating a critical role for Znf179 E3 ligase in maintaining the steady-state turnover rate of TDP-43 protein and preventing pathological TDP-43 accumulation. On the other hand, in Znf179 knockout mice, the cytosolic puctate staining of TDP-43 inclusions in hippocampus and cortex was elevated compared to controls. In combined with the data showing accumulation of insoluble polyubiquitinated full-length and truncated TDP-43 aggregates, our findings suggest that Znf179 E3 ligase activity is highly correlated with TDP-43 neuropathy.

Ubiquitination is an energy consuming cascade mediated by E1 (ubiquitin activating enzyme), E2 (ubiquitin-conjugating enzyme) and E3 (ubiquitin ligase) enzymes that sequentially transfer the ubiquitin molecule to a final substrate, often in order to mark it for degradation [43]. Among these enzymes, E3 ubiquitin ligases are crucial for substrate specificity during ubiquitin-dependent proteolysis [44]. E3 ubiquitin ligases are divided into two classes: HECT (homologous to E6-AP terminus) and RING proteins [21, 45]. The enzymatic activity of RING E3 ligases can be monitored through autoubiquitination [21, 32]. Various RING E3 ligases, including the Znf179 E3 ligase we identified here, have members of the UbcH5 family as their cognate E2 conjugating enzyme [31, 46]. Here we found that Znf179 possesses E3 ligase activity and exhibits autoubiquitination in vitro and in vivo in the presence of the UbcH5 E2 conjugating enzymes (Figs. 1-2), which is similar to a previous study demonstrating that Znf179 is polyubiquitinated in the presence of UbcH5 (also called UBE2D) E2 family proteins in vitro [47].

Autoubiquitination is a feature of RING E3 ubiquitin ligases, which results in the degradation or functional alteration of the RING E3 ligase proteins, depending on the ubiquitin linked lysine residues [31]. For example, autoubiquitination of Mdm2 recruits E2 conjugating enzymes and enhances its E3 ligase activity to polyubiquitinate p53 [46]. In contrast to Mdm2, autoubiquitination of another RING E3 ligase, BCA2, decreases its own protein stability and promotes its degradation through the proteasome [31]. Here we found that the protein half-life of the Znf179-5A mutant (T_1/2_ = 6.1 h) is shorter than that of wild-type Znf179 (T_1/2_ = 11.2 h) (Additional file 3: Figure S2A), and the protein level of Znf179-5A mutant is slightly lower than that of wild-type Znf179 (Additional file 3: Figure S2B), suggesting that autoubiquitination of Znf179 may enhance its stability rather than target Znf179 for proteasomal or autophagic degradation. In contrast, the mutation of the lysine residues on the RING finger domain of the Znf179-5A mutant renders the protein unable to be autoubiquitinated and subsequently makes them more susceptible to become unstable and degraded. When we inhibited UPS with a specific inhibitor, MG132, or inhibited autophagy with 3-MA in N2a cells, the protein levels of wild-type Znf179 or mutant Znf179-5A were roughly unchanged (Additional file 3: Figure S2B). These results suggest that the non-ubiquitinated Znf179 is degraded through both proteasomal and autophagic-lysosomal pathways, while autoubiquitination of Znf179 not only enhances its E3 ligase activity, but also prevents its degradation and maintains its protein stability.

Despite its being discovered over 20 years ago, the comprehensive cellular and molecular function of Znf179 is largely unknown. However, its gene mutations and expressions have shown the correlation of Znf179 with pathological diseases. For example, it has been found that one copy of the Znf179 gene (17p11.2, the most recombination-prone region of the genome) is deleted in Smith–Magenis syndrome patients and the gene product might play a role in pathogenesis related to regulating neuronal differentiation [48]. In terms of its normal cellular function, our group first discovered that Znf179 gradually increases in the developing brain and is critical for neuronal differentiation in cultured cells [25], suggesting its important function during neurogenesis. Recently, a new role for Znf179 has been discovered as a dynamin family GTPase which localizes to endosome and is involved in regulating excitatory synapses and spine density [47]. In the mammalian brain, the hyperfine regulation of dendritic spinogenesis is indispensable for learning and memory. We found that many of the Znf179 interacting proteins identified by MS and IPA are involved in dendritic arborization and growth (Additional file 2: Figure S3), and the Znf179 transgenic mice have improved performance in learning and memory tests (data not shown), implying the pivotal role of Znf179 in the regulation of dendritic spinogenesis and learning/memory processing. These findings describing the cellular function of Znf179 thus suggests an essential role of Znf179 in neuronal physiology. In addition, TDP-43 has also been implicated to mediate spine formation and maturation during brain development [49]. TDP-43 is a negative regulator of protrusion/spine generation, and the elevated level of TDP-43 in TDP-43 Tg/FTLD-TDP mice, similar to FTLD-TDP patients, results in the loss of dendritic spines/protrusions. Accordingly, TDP-43 could be one of the downstream signals for Znf179 that coordinates with Znf179 to mediate neurogenesis during brain development. Accumulated TDP-43 aggregates observed in Znf179 knockout mice may interfere with proper dendritic spine formation leading to neuronal dysfunction. Deregulation of the Znf179-TDP-43 signaling cascade might be one of the causes in neurodegenerative disease with TDP-43 proteinopathies.

Most of the polyubiquitinated soluble proteins are degraded by the UPS through the 26S proteasome [50], while the ubiquitinated misfolded/aggregated proteins are typically removed by the autophagy-lysosomal pathway [51–53]. The ubiquitin-proteasome is important in maintaining protein homeostasis in a broad spectrum of cellular processes. Ubiquitin is a 76 amino acid peptide that contains seven internal Lys residues (Lys6, Lys11, Lys27, Lys29, Lys33, Lys48 and Lys63), and the polyubiquitination chains can be linked via any of these positions, forming at least seven different types of linkages between ubiquitin molecules. Polyubiquitin linkage through Lys48 or Lys11 generally targets substrates to proteasome degradation, while monoubiquitination, oligomeric Lys63 or other linked ubiquitin chains may direct protein subcellular localization, inclusion formation, autophahy-lysosomal degradation or protein functional alterations [54, 55]. Under normal conditions, soluble TDP-43 is mostly degraded by the proteasome [56–58], while insoluble aggregated TDP-43 is removed by autophagy-lysosome system [20, 59–62]. Previously, Parkin was identified as an E3 ligase for TDP-43 that mediates polyubiquitination at Lys48 and Lys63 and promotes the translocation of TDP-43 from nucleus to cytosol, where it forms a complex with HDAC6 [63]. Parkin-mediated TDP-43 ubiquitination did not induce autophagy or proteasome degradation of TDP-43 proteins, but instead, altered its subcellular localization and facilitated its cytosolic accumulation, suggesting that other ubiquitin ligases, rather than Parkin, mediate the degradation of TDP-43 proteins. Moreover, the consequences of Parkin-mediated TDP-43 ubiquitination on the characteristics of TDP-43 proteinopathy, including protein degradation, protein half-life, insoluble aggregate formation or other functional alterations, were not comprehensively discussed. Znf179-mediated TDP-43 polyubiquitination has a broad range of biological effects on TDP-43 proteinopathies. Here we found that Znf179-mediated TDP-43 polyubiquitination enhances the degradation of both endogenous and exogenous TDP-43, which possibly occurs via Lys48 linkage-related proteasome-dependent proteolysis. In addition, in Znf179 knockout brains, the absence of Znf179 led to increased TDP-43 inclusions, suggesting that Znf179 may also induce the Lys63-mediated autophagic clearance of ubiquitinated TDP-43 inclusions. Lys63-linked ubiquitination would facilitate the clearance of ubiquitinated proteins via the autophagic pathway, and promote the autophagic clearance of protein inclusions in various neurodegenerative diseases [64]. Our results suggest that Znf179-mediated TDP-43 polyubiquitination not only regulates the normal routes of TDP-43 degradation via the UPS, but also reduces insoluble TDP-43 aggregates by stimulating autophagic clearance routes, implying a critical role for Znf179 E3 ligase in attenuating TDP-43-related neuropathy.

Several groups have discovered that the impairment of proteasome/lysosome activity is caused by intracellular deposition of pathological ubiquitin-positive inclusion bodies in various chronic neurodegenerative disorders, such as Alzheimer’s, Parkinson’s and Huntington’s diseases as well as ALS [65–67]. The alteration, mutation, deficiency or malfunctioning of E3 ligase and UPS-related factors may lead to hereditary neurodegenerative diseases [68]. For example, the E3 ligase TRAF6 (tumor necrosis factor receptor-associated factor 6), associating with and ubiquitinating huntingtin protein, promotes huntingtin protein aggregate formation in Huntington’s disease [69]. Several E3 ubiquitin ligases and UPS-related factors have been deemed to be good therapeutic targets in neurodegenerative diseases [70–74]. In ALS and FTLD-U mice and patients, Znf179 and a number of ubiquitin-proteasome related genes are downregulated [26, 67]. The downregulation of Znf179 in FTLD-U and ALS patients may be accompanied by diminished ubiquitin-proteasome activity leading to pathological TDP-43 aggregate accumulation. Targeting of the Znf179 E3 ligase could thus be a potential therapeutic strategy for TDP-43-related neurodegenerative diseases.

## Conclusions

Here we propose that Znf179 is a neural specific E3 ligase that regulates ubiquitin-proteasome dependent degradation of TDP-43 protein and TDP-43-related proteinopathies. A better understanding of the function of the novel E3 ligase, Znf179, and the correlation between Znf179 and TDP-43 will provide a new therapeutic avenue for the ALS and FTLD-U patients in the future.

## Additional files

Additional file 1: **Figure S1.** (A) Analysis the microarray data from GEO (GDS717), Znf179 mRNA expression in the whole brain of Huntington’s diasease (HD) transgenic mice. (B) Analysis the microarray data from GEO (GSE10953), Znf179 mRNA expression in brain of amyothophic lateral sclerosis (ALS) transgenic mice. (TIF 1315 kb)

Additional file 2: **Figure S2.** (A) 293 T cells transfected with Flag-Znf179 or Flag-Znf179-5A mutant for 48 h were then treated with 10 μM MG132 or 10 mM 3-MA for 4 h. Total cell lysates were analyzed by Western blotting with anti-Znf179 and anti-β-actin antibodies. (B) Protein intensity were quantified by AlphaEase FC software and the statistic results were presented as mean ± s.e.m. of at least three independent experiments (*, *P* < 0.05). (TIF 833 kb)

Additional file 3: **Figure S3.** (A) Immunoprecipitated mice whole brain lysates with anti-Znf179 antibody from wild-type and Znf179 knockout mice were separated by SDS-PAGE (4–20% polyacrylamide gel). Protein lands corresponding to immuno-positive bands were excised from gels (a, b, c and d bands), and analyzed with mass spectrometer. (B and C) Potential networks and involved subcellular pathways of identified proteins were further analyzed by IPA. (TIF 2176 kb)

## Abbreviations

ALS: amyotrophic lateral sclerosis
FTLD-U: frontotemporal lobar degeneration
HECT: homologous to E6-AP terminus
MS: mass spectrometry
RING: Really Interesting New Gene
TDP-43: 43 kDa nuclear protein TAR DNA-binding protein
UPS: ubiquitin proteasome system
