# Supplementary figures and images for: Znf179 E3 ligase-mediated TDP-43 polyubiquitination is involved in TDP-43- ubiquitinated inclusions (UBI) (+)-related neurodegenerative pathology
Source: J Biomed Sci. 2018 Nov 8;25:76. doi: 10.1186/s12929-018-0479-4 (PMC6223059; doi:10.1186/s12929-018-0479-4)

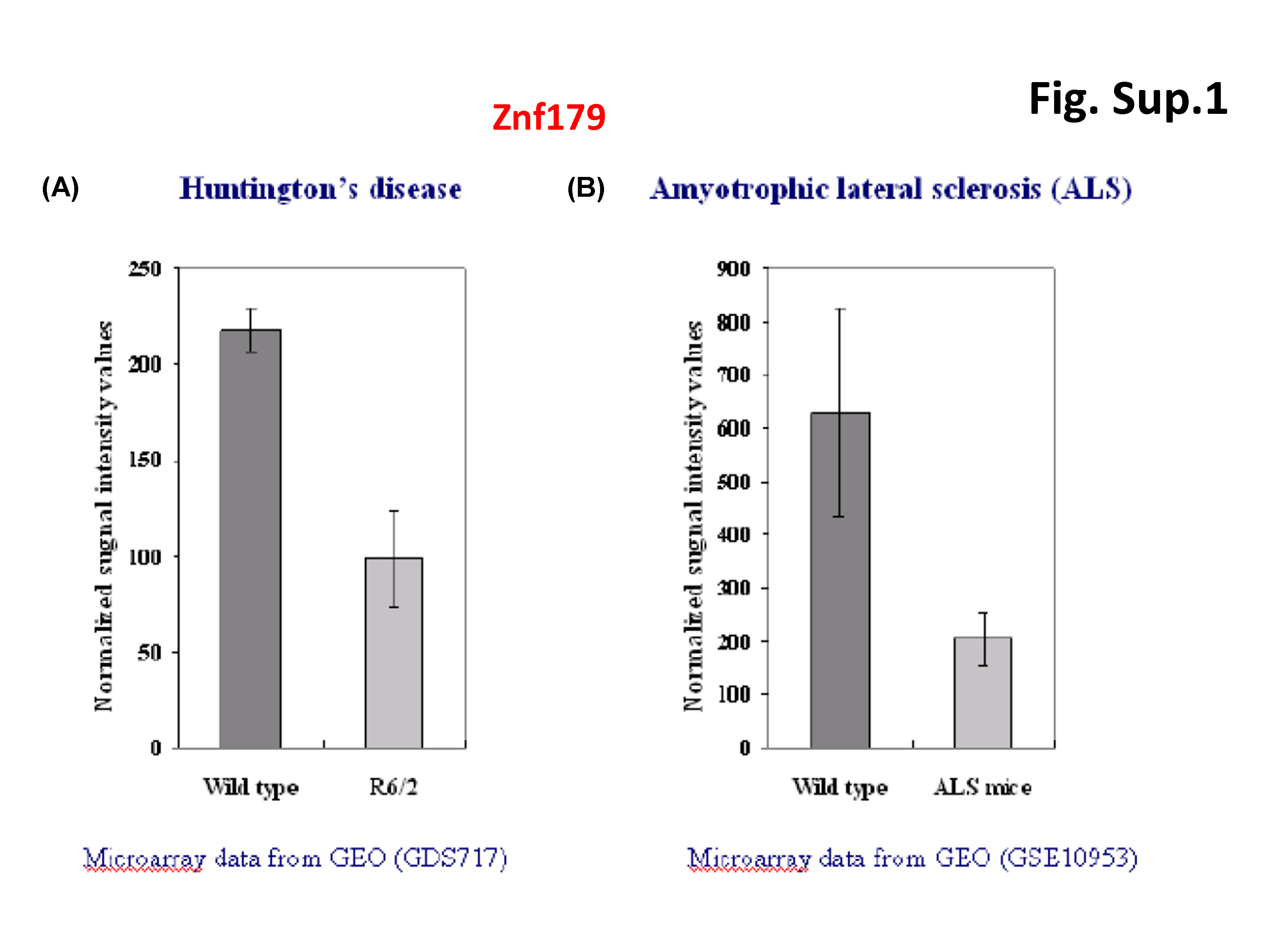

Supplement: Supplementary file 1 — Figure S1. (A) Analysis the microarray data from GEO (GDS717), Znf179 mRNA expression in the whole brain of Huntington’s diasease (HD) transgenic mice. (B) Analysis the microarray data from GEO (GSE10953), Znf179 mRNA expression in brain of amyothophic lateral sclerosis (ALS) transgenic mice. (TIF 1315 kb) [file 12929_2018_479_MOESM1_ESM.tif]

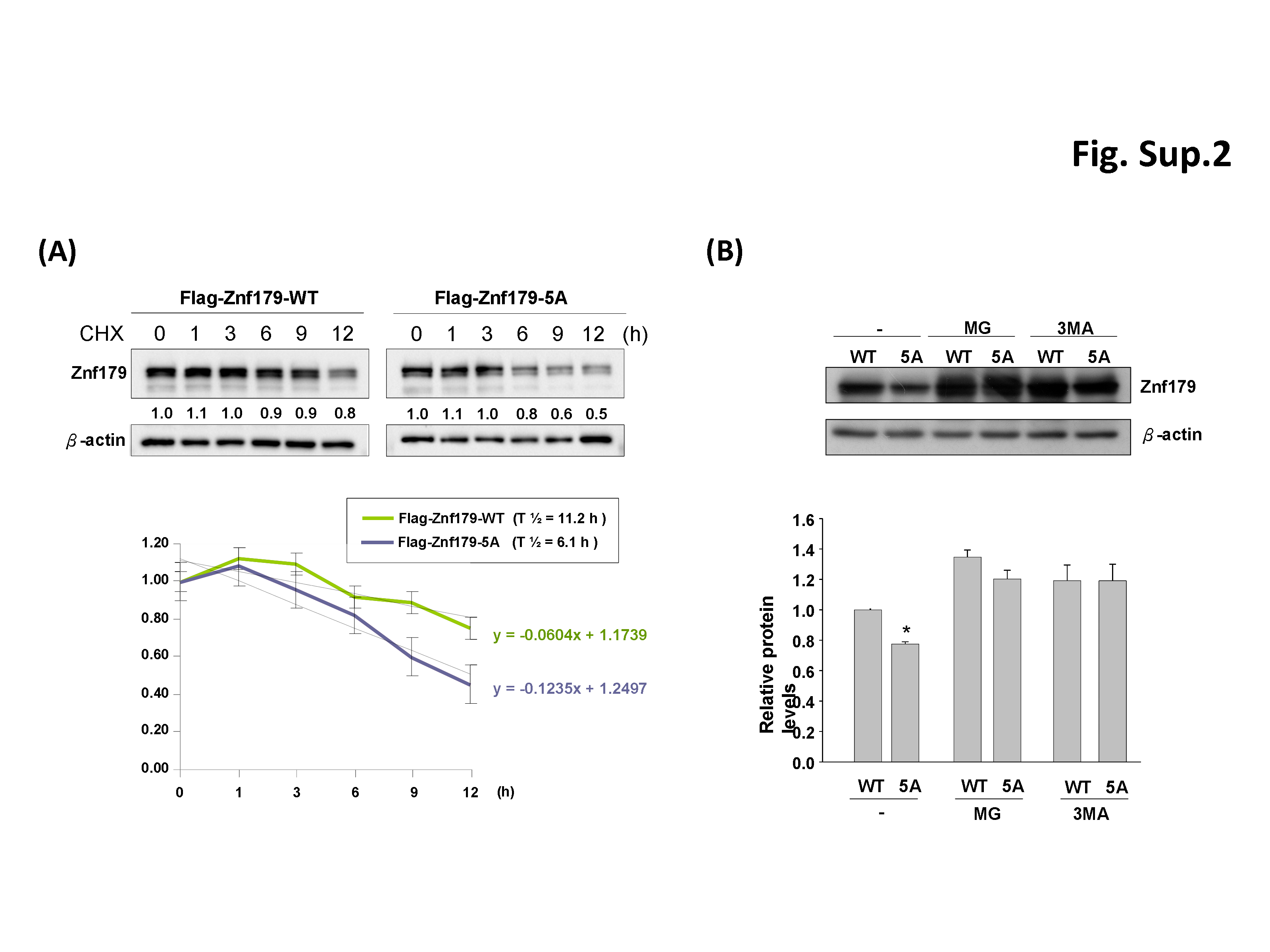

Supplement: Supplementary file 2 — Figure S2. (A) 293 T cells transfected with Flag-Znf179 or Flag-Znf179-5A mutant for 48 h were then treated with 10 μM MG132 or 10 mM 3-MA for 4 h. Total cell lysates were analyzed by Western blotting with anti-Znf179 and anti-β-actin antibodies. (B) Protein intensity were quantified by AlphaEase FC software and the statistic results were presented as mean ± s.e.m. of at least three independent experiments (*, P < 0.05). (TIF 833 kb) [file 12929_2018_479_MOESM2_ESM.tif]

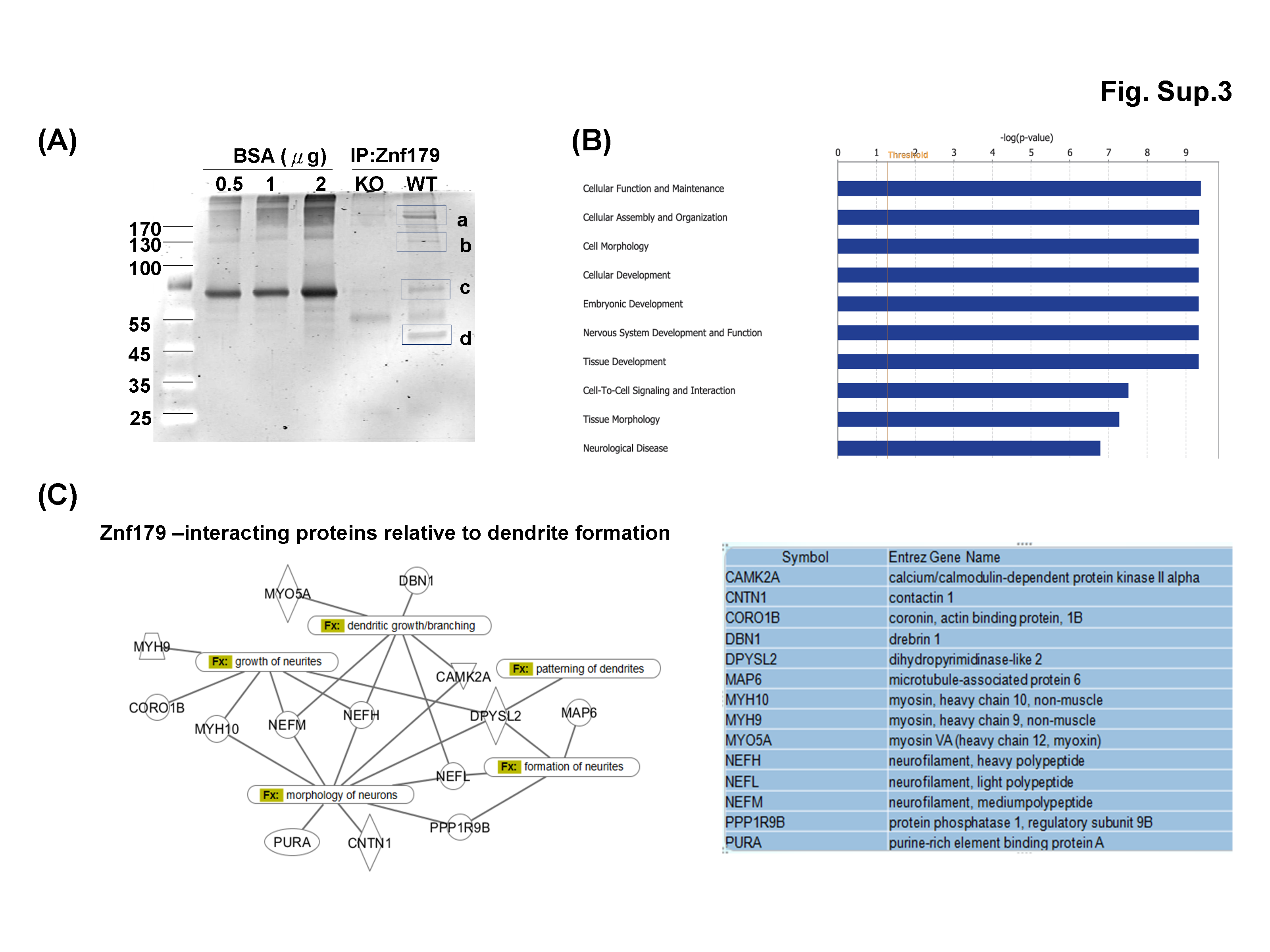

Supplement: Supplementary file 3 — Figure S3. (A) Immunoprecipitated mice whole brain lysates with anti-Znf179 antibody from wild-type and Znf179 knockout mice were separated by SDS-PAGE (4–20% polyacrylamide gel). Protein lands corresponding to immuno-positive bands were excised from gels (a, b, c and d bands), and analyzed with mass spectrometer. (B and C) Potential networks and involved subcellular pathways of identified proteins were further analyzed by IPA. (TIF 2176 kb) [file 12929_2018_479_MOESM3_ESM.tif]
